# Spatial distribution of metabolites in processing *Ziziphi Spinosae Semen* as revealed by matrix-assisted laser desorption/ionization mass spectrometry imaging

**DOI:** 10.1038/s41598-024-61500-w

**Published:** 2024-07-03

**Authors:** Donglai Ma, Mengwei Zhao, Haochuan Guo, Lili Wang, Yage Li, Shinong Yuan, Yuping Yan, Yuguang Zheng, Xian Gu, Yongxing Song, Xiaowei Han, Huigai Sun

**Affiliations:** 1 School of Pharmacy, Hebei University of Chinese Medicine, Shijiazhuang, 050200 China; 2 Traditional Chinese Medicine Processing Technology Innovation Center of Hebei Province, Shijiazhuang, 050200 China; 3 International Joint Research Center On Resource Utilization and Quality Evaluation of Traditional Chinese Medicine of Hebei Province, Shijiazhuang, 050091 China; 4 College of Chemistry and Chemical Engineering, Xingtai University, Xingtai, 054001 China

**Keywords:** Chemical biology, Plant sciences

## Abstract

*Ziziphi Spinosae Semen* (ZSS) is the first choice for the treatment of insomnia. This research aimed to reveal the spatial distribution of identifying quality markers of ZSS and to illustrate the metabolite quality characteristics of this herbal medicine. Here, we performed a matrix-assisted laser desorption/ionization-mass spectrometry imaging (MALDI-MSI) in situ to detect and image 33 metabolites in ZSS, including three saponins, six flavonoids, four alkaloids, eight fatty acids, and 12 amino acids. The MALDI images of the metabolites clearly showed the heterogeneous spatial distribution in different regions of ZSS tissues, such as the cotyledon, endosperm, and radicle. The distribution area of two saponins, six flavonoids, and three alkaloids increased significantly after the fried processing of ZSS. Based on the ion images, samples with different processing technologies were distinguished unambiguously by the pattern recognition method of orthogonal partial least squares discrimination analysis (OPLS-DA). Simultaneously, 23 major influencing components exerting higher ion intensities were identified as the potential quality markers of ZSS. Results obtained in the current research demonstrate that the processing of ZSS changes its content and distribution of the medicinal components. The analysis of MALDI-MSI provides a novel MS-based molecular imaging approach to investigate and monitor traditional medicinal plants.

## Introduction

*Ziziphi Spinosae Semen* (ZSS) is the dried mature seeds of *Ziziphus jujube* Mill. var. *spinosa* (Bunge) Hu ex H. F. Chou. It is well acknowledged for its sedative, hypnotic, antidepressant, anti-anxiety, and antioxidant effects, etc.^1–5^. ZSS is often used to treat central nervous system diseases, such as insomnia. It contains steroid saponins, flavonoids, alkaloids, fatty acids, and other metabolites^6–8^. spinosa and jujuboside A are the main components. Approximately 30% of the general population were reported to suffer from sleep disorders^9^. Thus, there is a growing demand for ZSS as a treatment for such disorders.

In addition, the ZSS also contains other ingredients. The terpenoid components in ZSS included jujuboside A, jujuboside B, and sanjoinenine, among which jujuboside A and jujuboside B are reported to have anti-hypertensive, anti-anxiety, anti-myocardial ischemia, and anti-neurasthenia activities, etc.^10,11^. The flavonoid components in ZSS included spinosin, 6‴-feruloylspinosin, vicenin-2,6″-p-coumaroylspinosin, and 6‴-sinapoylspinosin, which are some of the active components of the sedative, hypnotic, and antidepressant effects of ZSS^12–14^, and with important value in the quality control of ZSS. The alkaloid components in ZSS included (2S,3S)-N-[(1S)-1-Carbamoyl-3-methyl-butyl]-2-[[(2S)-2-dimethylamino-3-phenyl-propanoyl]amino]-3-(4-formylphenoxy)-4-methyl-valeramide (CMBDPM), nuciferine, amphibine D, and N-nornuciferine; when present in relatively low abundance, they are less toxic and safe to use^15,16^. These chemicals have sedative, hypnotic, anti-depressant, and anti-convulsant activities and can improve sleep. The most abundant component of ZSS was fatty acids with a content of up to 32%. More than 20 types of fatty acids have been isolated, most of which are unsaturated fatty acids^17,18^. Amino acids were essential for human survival, improving human immunity, and enhancing gastrointestinal absorption^19–21^. There were few reports on the amino acid composition of ZSS.

Studies on variation in metabolites of processed ZSS have mainly focused on flavonoids, saponins, and fatty acids^6,12^. Various compounds of raw ZSS (RZSS) and fried ZSS (FZSS) were identified by using ultra-high-performance liquid chromatography (UPLC) quadrupole time of flight (TOF) mass spectrometry (MS) technology combined with multivariate data analysis^22^. The relative quantitative analysis and content analysis of different compounds showed that the levels of six flavonoids, namely 6‴-feruloylspinosin, 6‴-p-coumaroylspinosin, 6‴-hetero-hydroxybenzoatespinosin, spinosin, 6‴-dihydrophaseoylspinosin, and 6‴-(-)-phaseoylspinosin, were relatively high in RZSS. The contents of jujuboside A and jujuboside B in 10 batches of RZSS and FZSS from different sources were analyzed by a high-performance liquid chromatography-diode array detector and evaporative light scattering detector which found the level of jujuboside A in FZSS was significantly higher than in RZSS^23^. The purpose of analyzing metabolites of RZSS and FZSS was to explore the scientific effects of processing and improving its quality standard.

Recently, HPLC, LC–MS, gas chromatography (GC)-MS, and other modern analytical techniques have been used to deeply analyze saponins, flavonoids, alkaloids, fatty acids, and other compounds in ZSS^24,25^. However, these technologies destroy the spatial distribution of metabolites in ZSS tissues during the pretreatment processes, such as extraction, purification, and enrichment, preventing genuine in situ analysis of metabolites.

In recent years, new molecular visualization techniques, such as matrix-assisted laser desorption/ionization mass spectrometry imaging (MALDI-MSI)^26–28^, can directly determine the content and spatial distribution of a large number of known or unknown compounds from a tissue surface. It is suitable for sample distribution analysis of complex systems. The large amount of information produced with this method also provides the possibility to solve problems in complex biological systems. It has been used to analyze the distribution of chemical components in plants, such as *Paeoniae Radix Alba*^29^, *Glycyrrhiza Radix et Rhizoma*^30^, *Ginkgo Folium*^31^, *Tetrastigma hemsleyanum* Diels and Gilg^32^, and *Aconiti Radix Cocta*^33^. This method was also used to explore the relationship between the accumulation of chemical components during plant growth and processing and their efficacy.

In this research, 2-mercaptobenzothiazole (2-MBT) was used as the MALDI matrix for the matrix coating on the surface of frozen sections of ZSS. The MALDI-MSI technology was used to characterize saponins, flavonoids, alkaloids, fatty acids, and amino acids in RZSS and FZSS. Multivariate statistical methods were used to screen the key difference materials related to RZSS and FZSS. The results of mass spectrometry imaging of RZSS and FZSS can help us to better understand the chemical changes of traditional Chinese medicine during processing.

## Results

### Morphological characteristics of ZSS

**Figure 1:**
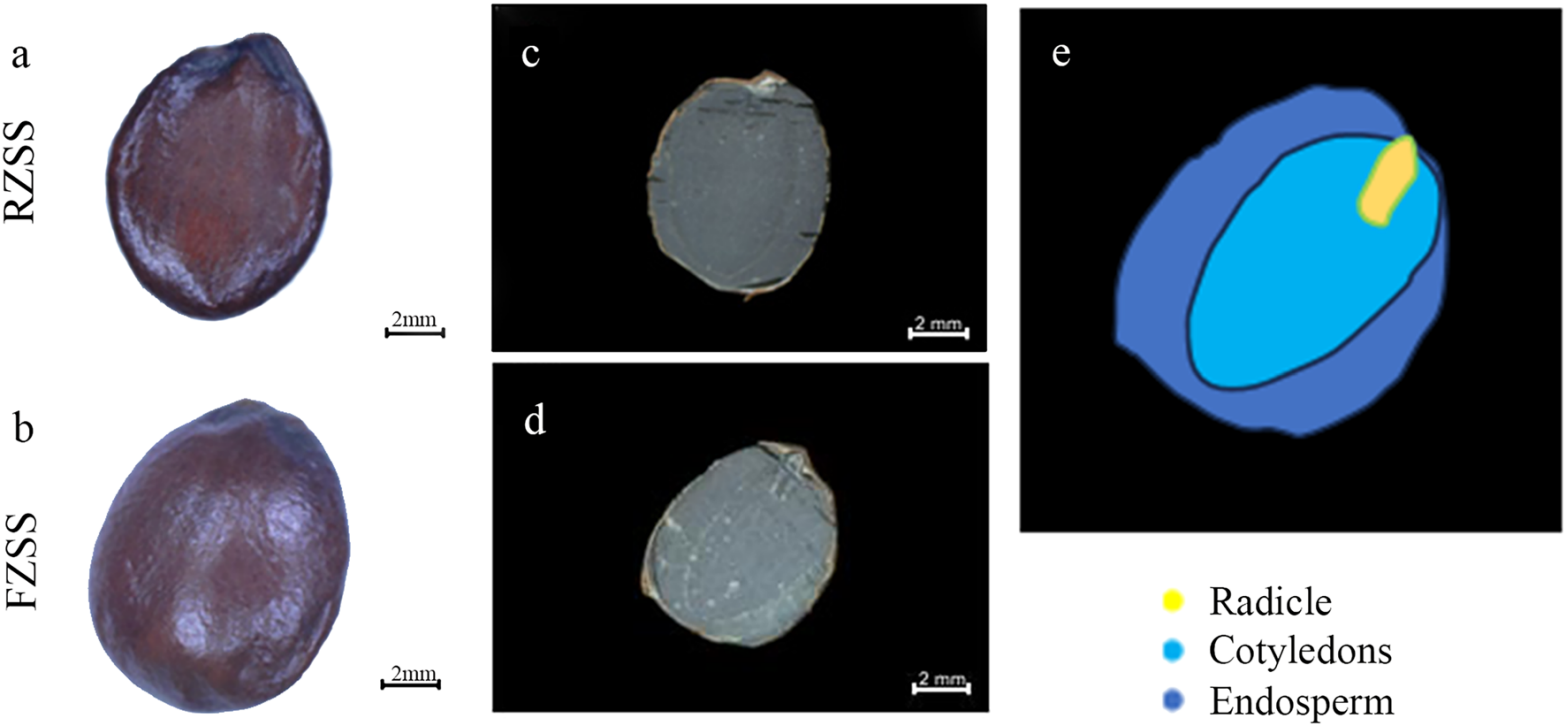
Morphological characteristics of ZSS (**a** and **b**), macroscopic features of transverse sections (**c** and **d**), and Schematic of ZSS (**e**).

The surface of ZSS was purplish red or purplish brown, smooth, and glossy, while the surface of FZSS was slightly bulging and slightly burnt (Fig. 1a,b). There was no significant difference in the length and diameter of RZSS and FZSS. Compared with the RZSS, the FZSS had a slightly burnt aroma. To further observe the microscopic characteristics of RZSS and FZSS (Fig. 1c,d). The internal structure of the cross-section from outside to inside is endosperm, radicle, and cotyledon (Fig. 1e).

### In situ metabolite profiling in RZSS and FZSS using MALDI-MSI

**Figure 2:**
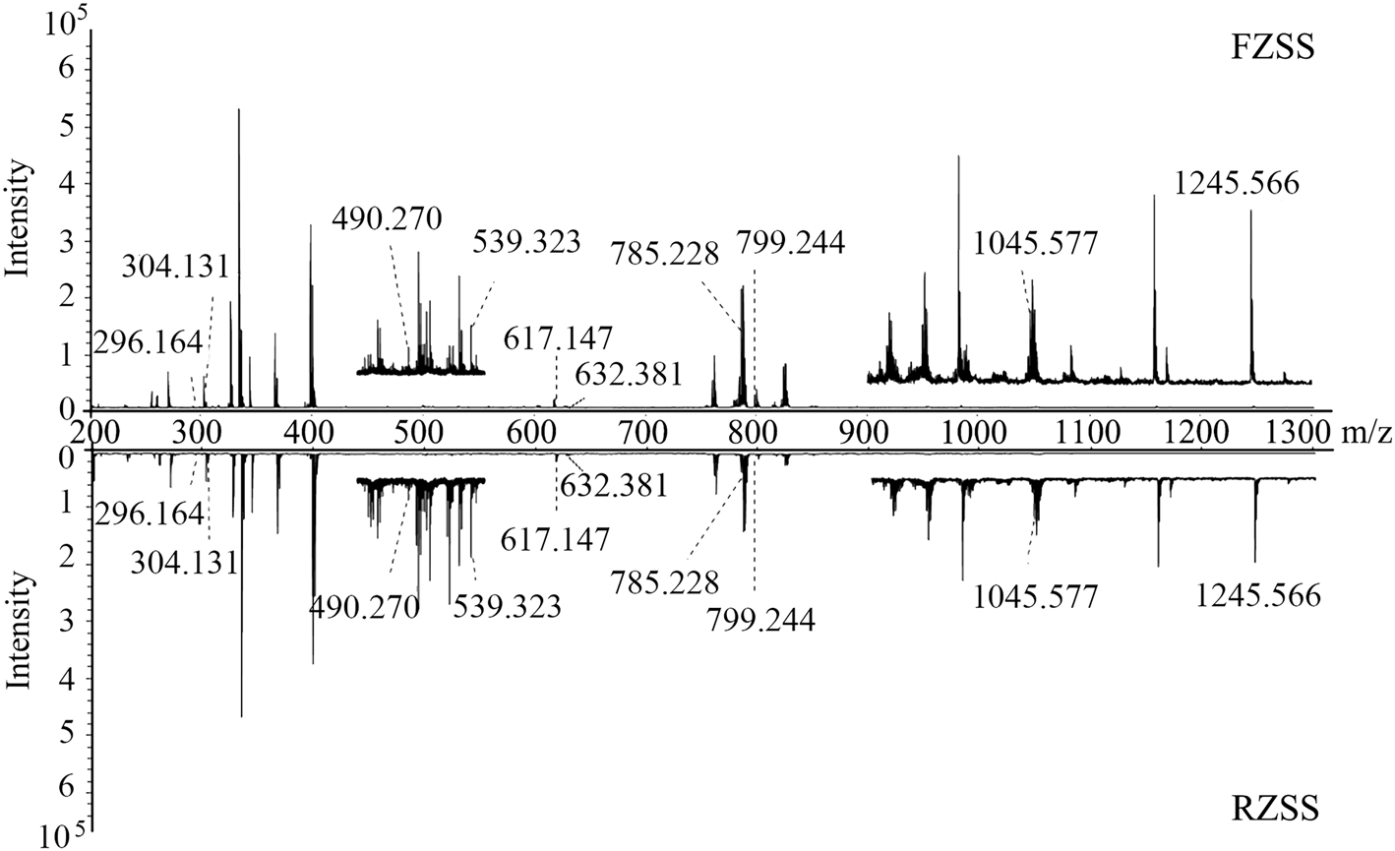
Comparison of mass spectra acquired from laser irradiation of tissue sections of RZSS and FZSS by (+) MALDI-TOFTOF MS using 2-MBT as the matrix.

A comprehensive metabolite profile for RZSS and FZSS in the positive-ion mode was generated by MALDI-MSI (Fig. 2). The positive ions were detected primarily in the *m/z* 100–400 and *m/z* 700–900 ranges. The assignment of the components was based on ion screening using the ZSS standards, literature, and other databases. The MALDI-MSI analysis allowed the detection of an on-tissue positive ion of 33 identified compounds, including terpenes, flavonoids, alkaloids, amino acids, and organic acids (see Supplementary Table S1).

### Detection of the spatial distribution of terpenoids in RZSS and FZSS using MALDI-MSI

**Figure 3:**
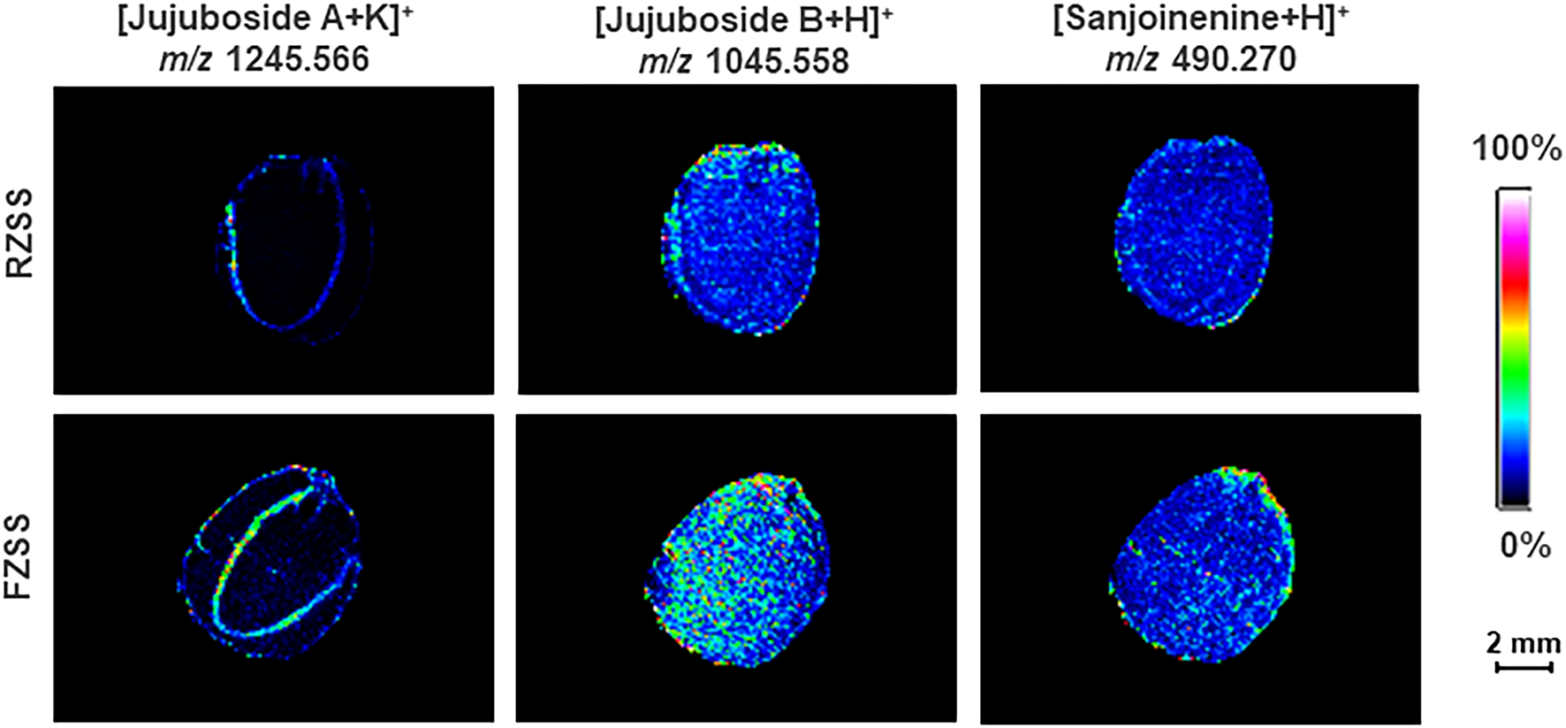
Selected ion maps of three terpenoids detected from RZSS and FZSS tissue sections by MALDI-TOFTOF MS in the positive-ion mode using 2-MBT as the matrix. A color scale from white to blue indicates a high-to-low sign.

The spatial distribution of metabolites of jujuboside A (*m/z* = 1245.566, [M + K]^+^), jujuboside B (*m/z* = 1045.558, [M + H]^+^), and sanjoinenine (*m/z* = 490.270, [M + H]^+^) were collected via MALDI imaging of the ZSS cross sections (Fig. 3) and were mainly distributed in the cotyledons and endosperm. Compared with RZSS anatomical images, jujuboside A and sanjoinenine in FZSS increased by 19.63% and 39.75%, respectively. Jujuboside B decreased by 4.74%.

### Detection of the spatial distribution of flavonoids in RZSS and FZSS using MALDI-MSI

**Figure 4:**
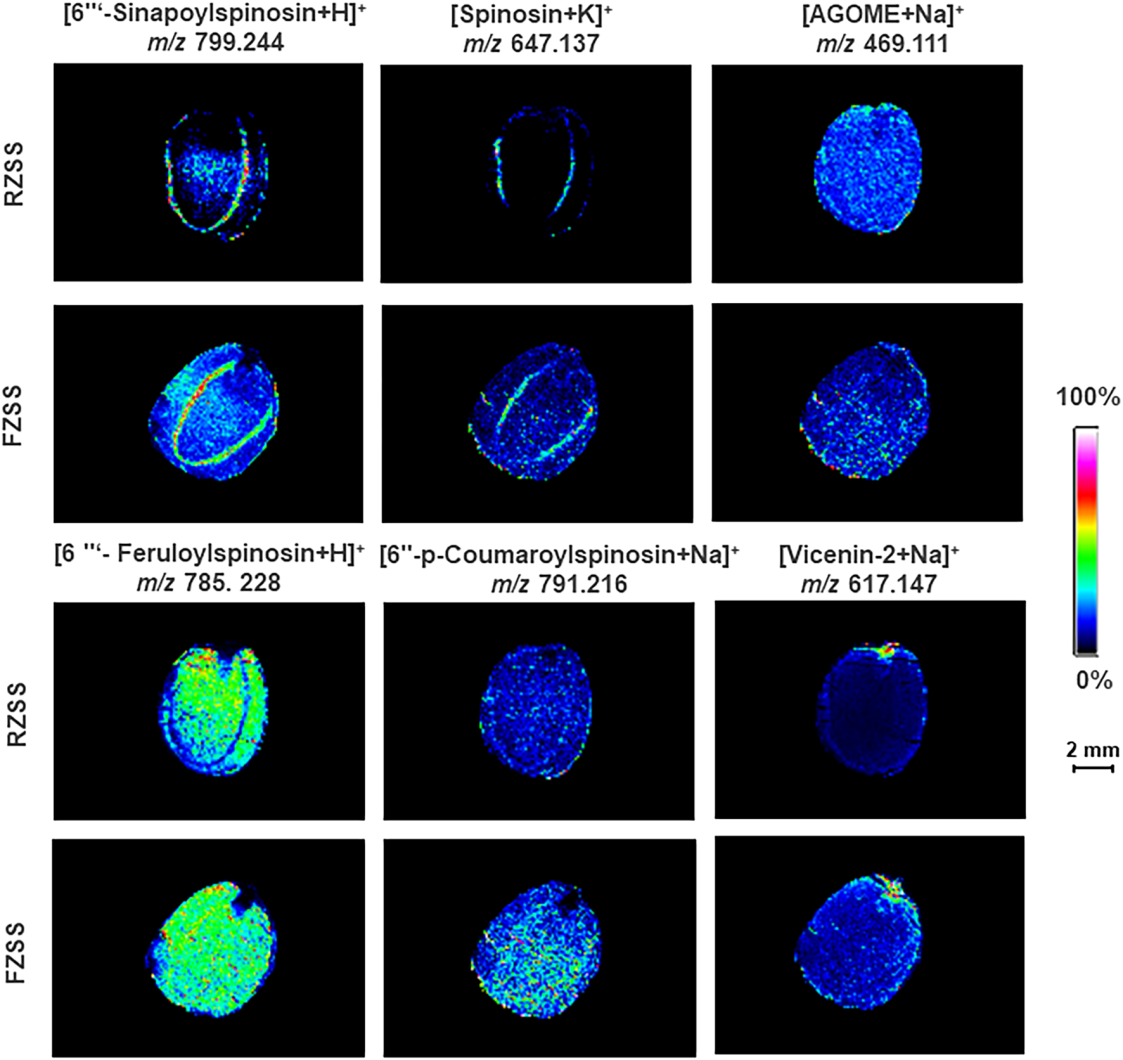
Selected ion maps of three terpenoids detected from RZSS and FZSS tissue sections by MALDI-TOFTOF MS in the positive-ion mode using 2-MBT as the matrix. A color scale from white to blue indicates a high- to low-signal intensity.

The metabolites of spinosin (*m/z* = 647.137, [M + K]^+^), 6‴-feruloylspinosin (*m/z* = 785.288, [M + H]^+^), Apigenin-6-glucosyl-7-O-methyl ether (AGOME) (*m/z* = 469.111, [M + Na]^+^), vicenin-2 (*m/z* = 617.147, [M + Na]^+^), 6″-p-coumaroylspinosin (*m/z* = 791.216, [M + Na]^+^), and 6‴-sinapoylspinosin (*m/z* = 799.244, [M + H]^+^) were mainly distributed in the cotyledons and the radicle (Fig. 4). Compared with RZSS anatomical images, the levels of spinosin, 6‴-feruloylspinosin, AGOME, vicenin-2,6″-p-coumaroylspinosin, and 6‴-sinapoylspinosin in FZSS decreased by 165.72%, 51.60%, 80.32%, 3.44%, 33.92%, and 201.23%, respectively.

### Detection of the spatial distribution of alkaloids in RZSS and FZSS using MALDI-MSI

**Figure 5:**
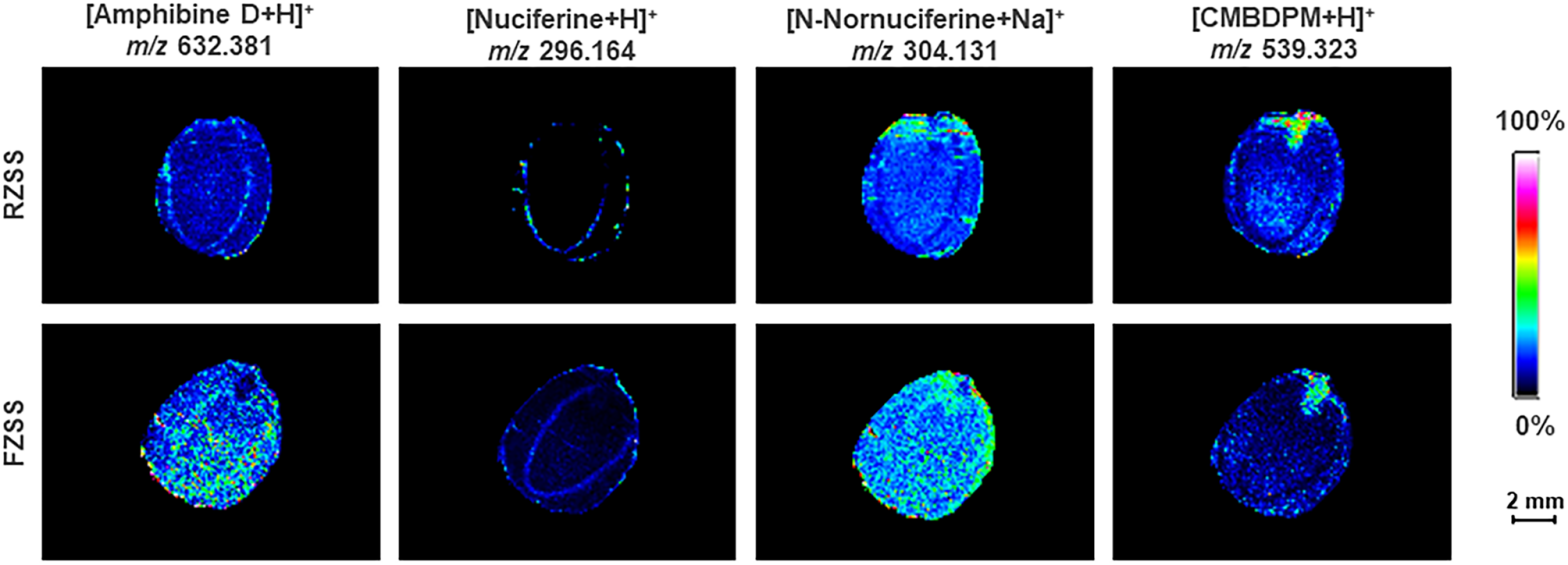
Ion maps of four alkaloids detected from RZSS and FZSS tissue sections by MALDI-TOFTOF MS in the positive-ion mode using 2-MBT as the matrix. A color scale from white to blue indicates a high- to low-signal intensity.

The spatial distribution information of four metabolites of (2S,3S)-N-[(1S)-1-Carbamoyl-3-methyl-butyl]-2-[[(2S)-2-dimethylamino-3-phenyl-propanoyl]amino]-3-(4-formylphenoxy)-4-methyl-valeramide (CMBDPM) (*m/z* = 539.323, [M + H]^+^), nuciferine (*m/z* = 296.164, [M + H]^+^), amphibine D (*m/z* = 632.381, [M + H]^+^), and N-nornuciferine (*m/z* = 304.131, [M + Na]^+^), were collected via MALDI imaging of the cross-section of ZSS (Fig. 5). They were mainly distributed in the cotyledons and endosperm. Compared with RZSS anatomical images, nuciferine, amphibine D, N-nornuciferine, and CMBDPM in FZSS decreased by 13.67%, 34.89%, 5.36%, and 13.64%, respectively.

### Detection of the spatial distribution of fatty acids in RZSS and FZSS using MALDI-MSI

**Figure 6:**
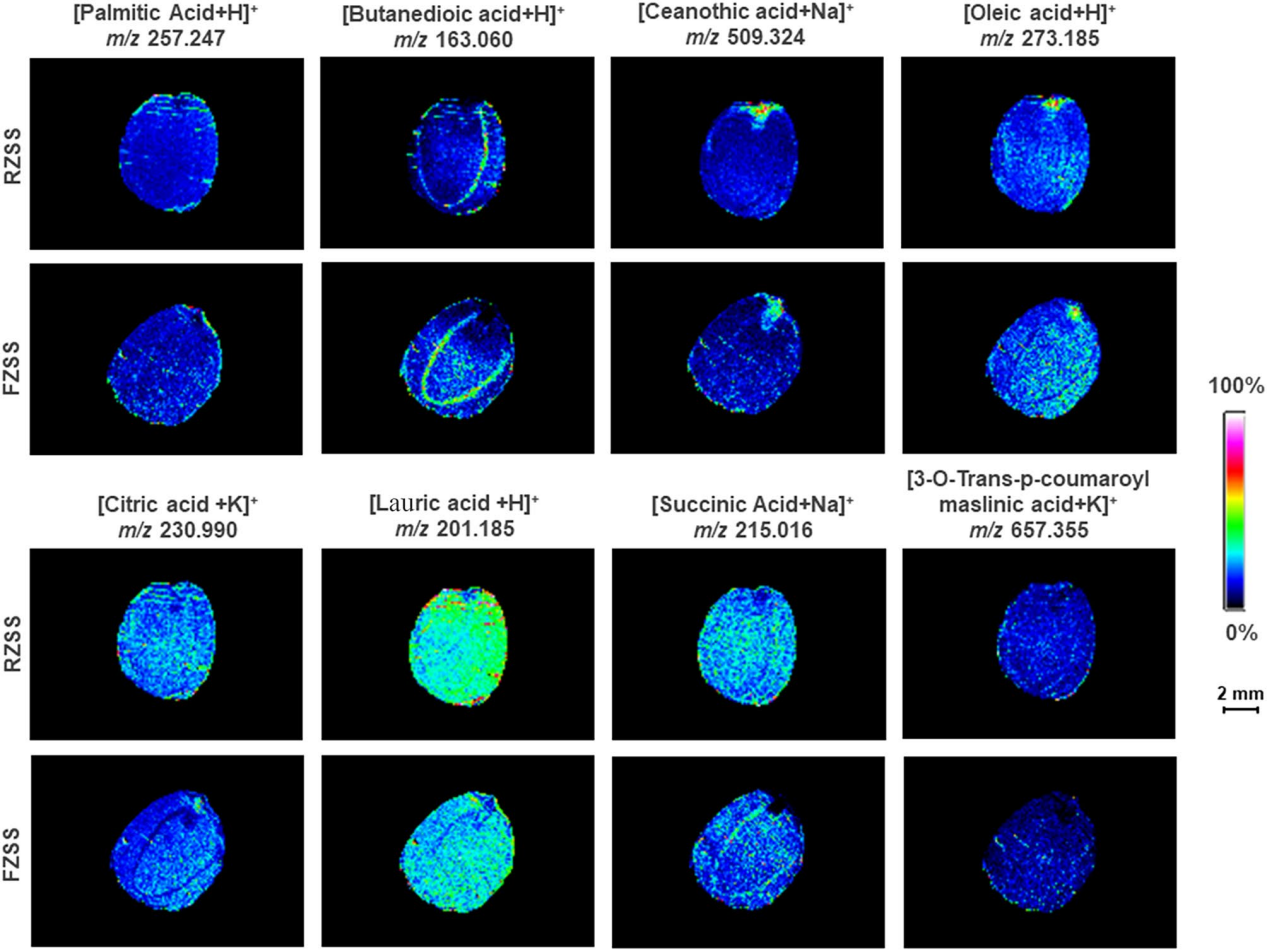
Ion maps of eight fatty acids detected from RZSS and FZSS tissue sections using MALDI-TOFTOF MS in the positive-ion mode using 2-MBT as the matrix. A color scale from white to blue indicates a high to low signal intensity.

The spatial distribution of eight metabolites, palmitic acid (*m/z* = 257.247, [M + H]^+^), oleic acid (*m/z* = 273.185, [M + H]^+^), succinic acid (*m/z* = 215.016, [M + Na]^+^), citric acid (*m/z* = 230.990, [M + K]^+^), 3-O-trans-p-coumaroylmaslinic acid (*m/z* = 657.355, [M + K]^+^), ceanothic acid(*m/z* = 509.324, [M + Na]^+^), butanedioic acid (*m/z* = 163.060, [M + H]^+^), and lauric acid (*m/z* = 201.185, [M + H]^+^), was collected using MALDI imaging of the cross-section of ZSS (Fig. 6). These metabolites were mainly distributed in the cotyledons and radicle. Compared with RZSS anatomical images, the levels of palmitic acid, butanedioic acid, and ceanothic acid in FZSS increased by 15.80%, 28.75%, and 6.07%, respectively; and oleic acid, citric acid, lauric acid, succinic acid, and 3-O-trans-p-coumaroyl maslinic acid decreased by 52.83%, 55.70%, 16.84%, 3.74%, and 1.38%, respectively (Fig. 6).

### Detection of the spatial distribution of amino acids in RZSS and FZSS using MALDI-MSI

**Figure 7:**
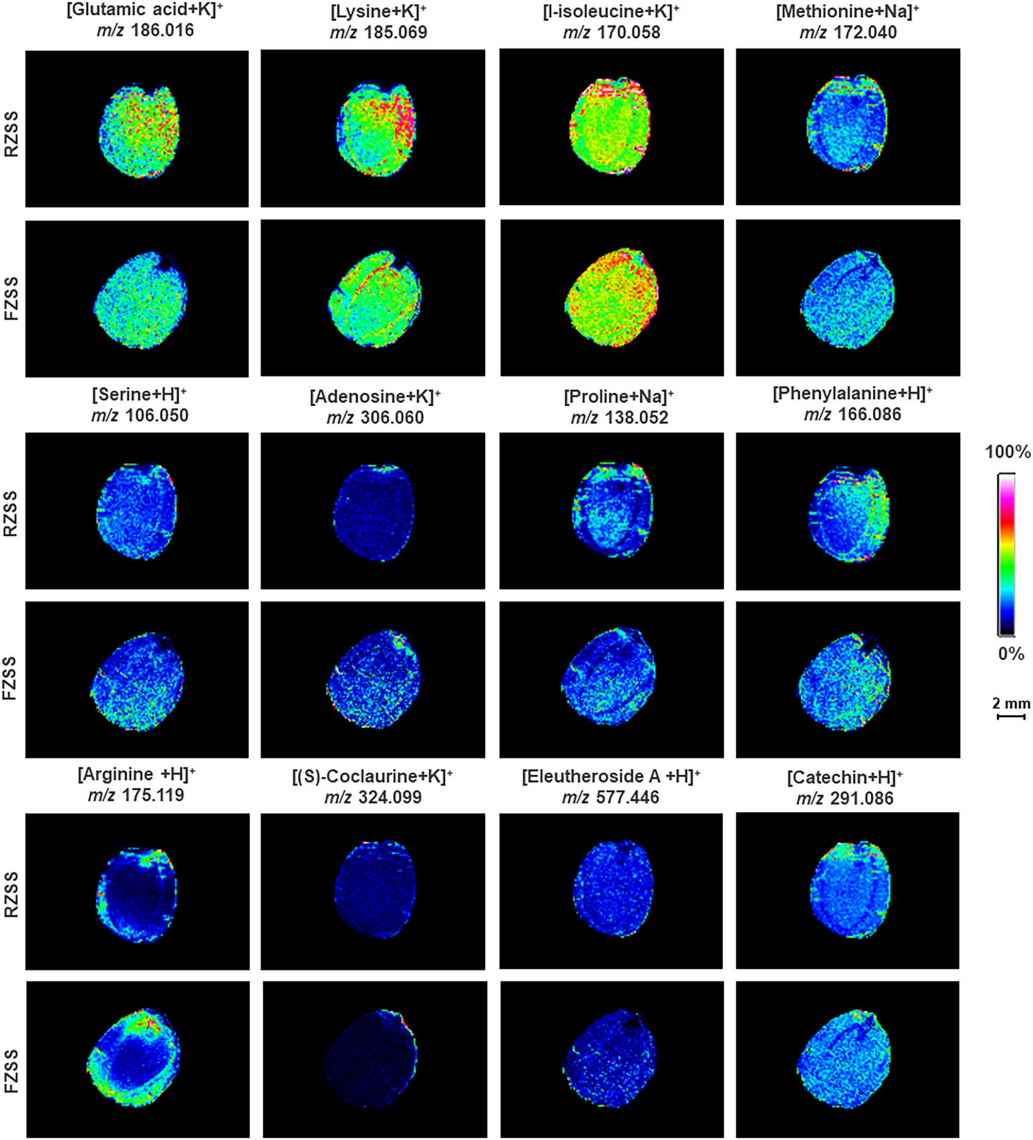
Ion maps of 12 amino acids detected from RZSS and FZSS tissue sections by MALDI-TOFTOF MS in the positive-ion mode using 2-MBT as the matrix. A color scale from white to blue indicates a high to low signal intensity.

Using MALDI acquisition, we determined the distribution of twelve metabolites, including glutamic acid (*m/z* = 186.016, [M + K]^+^), proline (*m/z* = 138.052, [M + Na]^+^), lysine (*m/z* = 185.069, [M + K]^+^), phenylalanine (*m/z* = 166.086, [M + H]^+^), methionine (*m/z* = 172.040, [M + Na]^+^), L-isoleucine (*m/z* = 170.058, [M + K]^+^), serine (*m/z* = 106.050, [M + H]^+^), arginine (*m/z* = 175.119, [M + H]^+^), (S)-coclaurine (*m/z* = 324.099, [M + K]^+^), eleutheroside A (*m/z* = 577.446, [M + H]^+^), adenosine (*m/z* = 306.060, [M + K]^+^), and catechin (*m/z* = 291.086, [M + H]^+^), in the cotyledons and endosperm (Fig. 7). Compared with RZSS anatomical images, the levels of glutamic acid, proline, lysine, phenylalanine, methionine, L-isoleucine and serine in FZSS were slightly increased. In contrast, arginine, (S)-coclaurine, eleutheroside A, adenosine, and catechin increased by 710.97%, 70.83%, 61.93%, 10.31%, and 6.66%, respectively.

### Potential quality-associated markers for RZSS and FZSS discovered by MALDI-MSI combined with OPLS-DA

To explore the potential of mass spectrometry imaging in quality illustration of herbal medicine, representative RZSS and FZSS possessing 5 categories of quality characters were analyzed by MALDI-MS. Although the spatial distribution pattern of metabolites was born with some similarities in different categories of samples, their relative signal intensity in smaller tissues varied. The pattern recognition method OPLS-DA can distinguish among RZSS and FZSS groups and, simultaneously, identify the major contributing components. The OPLS-DA models based on MSI data of the cross-section within the whole spectral ranges (*m/z* 100–1200) under RZSS and FZSS groups were further established to clarify the different components produced in the fried processing. PCA and OPLS-DA were performed to determine the difference in compounds between RZSS and FZSS (see Supplementary Fig. S1a and b). Their independent variable model parameter (R^2^X) and dependent variable model parameter (R^2^Y) were both greater than 0.5, indicating that the model has good stability and prediction ability. The result of the permutation test plot showed that the model did not overfit, and had good prediction ability (see Supplementary Fig. S1c). Normally, the very important projection (VIP) reflects the contribution of each metabolic component to the group. The VIP values of proline, (S)-coclaurine, lysine, phenylalanine, glutamic acid, arginine, L-isoleucine, butanedioic acid, oleic acid, lauric acid, methionine, citric acid, 6‴-sinapoylspinosin, palmitic acid, AGOME, catechin, spinosin, 6‴-feruloylspinosin, eleutheroside A, 6″-p-coumaroylspinosin, N-nornuciferine, amphibine D, serine, adenosine, and nuciferine were all greater than 1 (see Supplementary Fig. S1d). Therefore, these components could be used as potential quality-associated markers to distinguish RZSS and FZSS.

## Discussion

MALDI-MSI is an innovative molecular imaging technology that could simultaneously image various endogenous or exogenous molecules in biological tissue sections without specific labeling and complex sample pretreatment^34,35^. It offers high resolution, high sensitivity, high specificity, a broad mass range, and a rapid experimental period. It enables the acquisition of relative abundance information on compounds in situ, thereby providing spatial information^26^. In our research, MALDI-MSI was utilized to directly analyze the spatial distribution of 33 metabolites in RZSS and FZSS, including saponins, flavonoids, alkalis, amino acids, fatty acids, and organic acids.

The accumulation of bioactive compounds in ZSS contributes to its sedative, anti-convulsant, and anxiolytic activities^3^. These metabolites also serve as the material basis for seed life processes and exhibit significant regional differences within the seeds^36^. The results of this research indicated that saponins were concentrated in the cotyledons and endosperm of ZSS, while flavonoids exhibit higher levels in the cotyledons and radicle, alkaloids were primarily located in the cotyledons and endosperm, amino acids showed significantly higher concentrations in the cotyledons and radicle, and fatty acid compounds were distributed across different tissue regions of ZSS.

FZSS is a fried product of RZSS that undergoes heating and frying^37^, resulting in significant differences in various metabolites compared to RZSS^28^. Specifically, FZSS contains higher levels of saponins jujuboside A and sanjoinenine, the fatty acid-like butanedioic acid, amino acids including arginine, (S)-coclaurine, and eleutheroside A, while exhibiting lower levels of flavonoids such as spinosin, 6‴-feruloylspinosin, AGOME, 6″-p-coumaroylspinosin, and 6‴-sinapoylspinosin, and alkaloids like nuciferine and amphibine D. It can be seen that by heating and frying can increase saponin levels and decrease flavonoids levels in RZSS. Jujuboside A is one of the most important components for the treatment of insomnia. Heating and frying contribute to the increase and dissolution of jujuboside in FZSS. This is why the FZSS has a stronger calming role. Heating and frying will increase most of the fat oil and amino acid levels, which will be more suitable for insomnia people with weak constitutions.

The sedative and hypnotic effects of ZSS are primarily attributed to flavonoids and saponins, with jujuboside A and spinosin being the most distinctive pharmacodynamic components that serve as quality markers for ZSS^38^. Jujuboside A was found to be the predominant compound in FZSS. Further analysis found that sanjoinenine was more abundant in FZSS than in RZSS, which is consistent with previous research by HPLC and GC studies^39^. Moreover, FZSS exhibited superior sedative properties compared to RZSS, which could be explained from the perspective of spatial chemistry^40^. Through MALDI-MSI, we were able to observe the spatial distribution difference of the main components of FZSS and RZSS more intuitively, which increased our understanding of the technical means of traditional Chinese medicine processing.

## Conclusion

We have visualized the spatial and temporal distribution patterns of 33 metabolites in processed ZSS, which provides valuable data for a better understanding of the sedative and hypnotic activities of ZSS. Through MALDI-MSI analysis, we have identified saponin, flavonoid, alkaloid, and fatty acid components with specific medicinal activities in ZSS, providing new insights into its medicinal properties and potential applications. Our findings revealed that the peak intensity increased in a tissue-specific localized manner for saponins, flavonoids, and alkaloids, but most amino acids and fatty acids exhibited decreased peak intensities in RZSS. The peak intensities of most of the amino acids and fatty acids decreased. The spatial signals of metabolites in processed ZSS were labeled to establish a scientific basis for the quality evaluation of ZSS.

## Materials and methods

### Chemicals and plant materials

The LC–MS grade methanol, ethanol, acetonitrile, formic acid, 0.1% (w/v) poly(L-lysine) hydrobromide solution, and gelatin were obtained from Sigma-Aldrich (Shanghai, China). The analytical grade ammonium acetate, chloral hydrate, phloroglucinol, iodine, sulfuric acid, and ethanol were obtained from Sinopharm Chemical Reagent Co., Ltd. (Beijing, China). The optimum cutting temperature compound was obtained from Leica (Nussloch, Germany). Water was purified with a Milli Q filtration system (Millipore, Bedford, MA, USA). RZSS and FZSS were provided by the Shijiazhuang Yiling Pharmaceutical Co., Ltd., Hebei Province, China, in August 2021. RZSS and FZSS all are in accordance with the Pharmacopoeia of the People’s Republic of China (Volume I)^38^.

### Preparation of low molecular weight standard compounds

Standard solutions of each saponin, flavonoid, alkaloid, fatty acid, amino acid, and other low molecular weight compound, including jujuboside A, jujuboside B, sanjoinenine, spinosin, 6‴-feruloylspinosin, AGOME, vicenin-2, nuciferine, amphibine D, N-nornuciferine, glutamic acid, proline, lysine, phenylalanine, methionine, L-isoleucine, arginine, serine, (S)-coclaurine, eleutheroside A, adenosine, catechin, palmitic acid, oleic acid, succinic acid, citric acid, 3-O-trans-p-coumaroyl maslinic acid, ceanothic acid, butanedioic acid, and lauric acid, were prepared at a concentration of 1 mmol in deionized water for MALDI-TOF MSI detection. To prepare the mixed standard solution, 30 low molecular weight compounds were dissolved in deionized water at a concentration of 10 mM, and diluted with deionized water to desired concentrations. One microliter of each standard solution was then spotted onto an AnchorChip target plate for the MALDI-TOF MS detection.

### Tissue sectioning

The RZSS and FZSS tissues were cryo-sectioned at − 20 °C into 20-μm thick slices in a Leica CM1860 cryostat (Leica Microsystems, Wetzlar, Germany). The serial tissue slices were then immediately thaw-mounted on the conductive sides of indium tin oxide-coated microscope glass slides (Bruker Daltonics, Billerica, MA, USA).

### Matrix coating

MALDI matrices were coated using an ImagePrep electronic matrix sprayer (Bruker Daltonics, Bremen, Germany). The matrix for detection was 12 mg/mL 2-MBT in methanol: water (4:1, v/v) containing 0.2% trifluoroacetic acid. The matrix spraying was conducted according to the spray method provided by the instrument manufacturer with some modifications.^41,42^ During the matrix spraying by the ImagePrep sprayer, the incubation time was 30 s, the wetness was 40%, and other parameters were set as default for the standard method. Sprayed tissue sections were prepared for the MALDI-MSI analysis, and an Epson Perfection V550 Photo Scanner (Epson (China) Co., Ltd., Beijing, China) was used to take optical images of the tissue sections.

### MALDI-MS

All the profiling and imaging experiments were performed using an Autoflex Speed MALDI-TOF/TOF–MS (Bruker Daltonics, Billerica, MA, USA). The MALDI source was equipped with a 2000 Hz solid-state Smartbeam Nd: YAG UV laser (355 nm, Azura Laser AG, Bremen, Germany). Mass spectra were acquired over a mass range of *m/z* 100–2000 in a positive-ion mode with broadband detection. The mass spectra were recorded from 20 laser scans to acquire MALDI-MS profiling data. Each scan was accumulated from 500 laser shots. For imaging data acquisition, 100 µm laser raster step sizes were used to detect endogenous low molecular weight compounds in the RZSS and FZSS tissue sections, and each scan (pixel) was accumulated from 500 laser shots.

**Figure 8:**
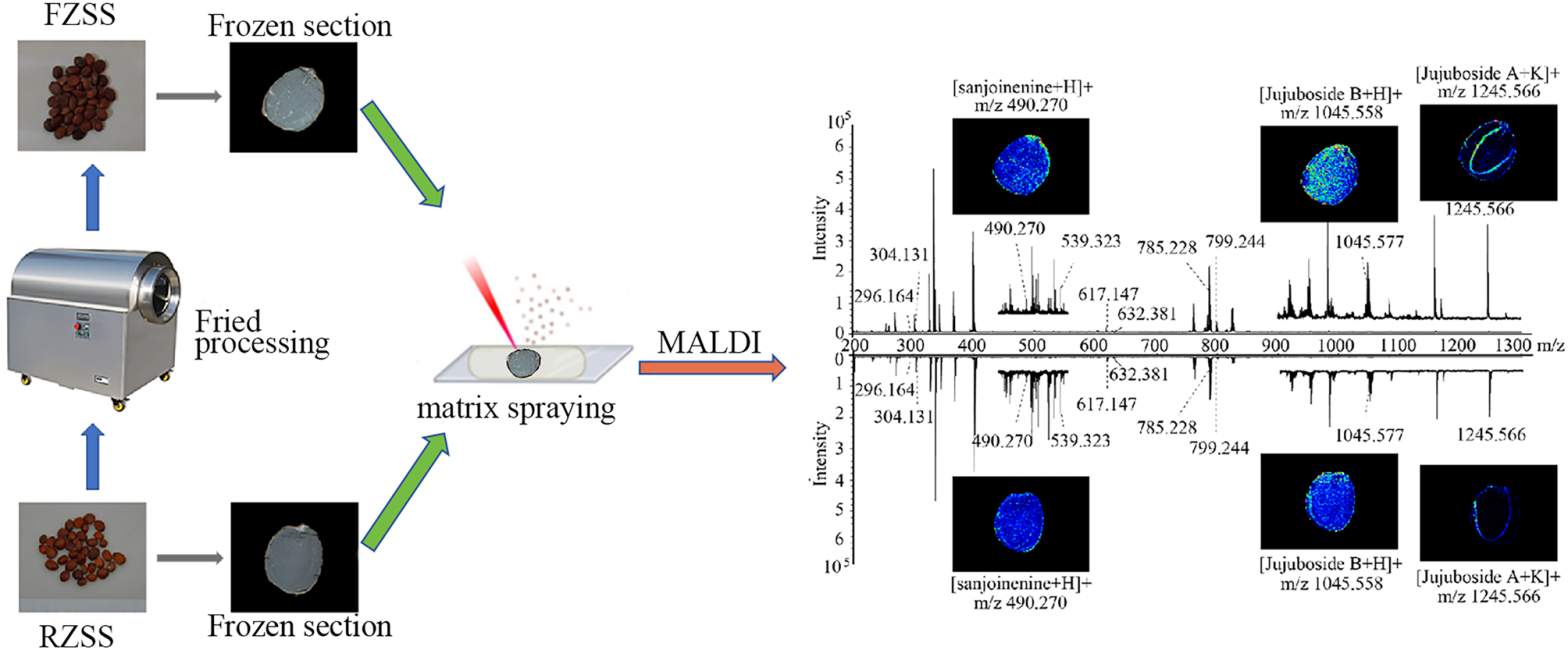
Workflow for MALDI on FZSS and RZSS.

Based on Bruker’s FlexImaging 4.1 software (https://www.bruker.com/zh/services/software-downloads.html), a correction pen was used to mark the “teaching points” (generally three points) around a tissue section for the correct positioning of the UV laser for spectral acquisition. The MALDI-TOF mass spectra were processed with the total ion current normalization, and the signal intensity of each imaging data was represented as the normalized intensity (Fig. 8).

### Data analysis

After data collection using MALDI-TOF-MSI, spectral files were processed using FlexAnalysis 3.4 software (Bruker Daltonics GmbH, Bremen, Germany) of formalin-fixed paraffin-embedded tissue by the MALDI imaging mass spectrometry. In the measured area of each sample, cotyledon, radicle, endosperm, and other tissues were defined according to the botanical structure of ZSS. Regions of interest containing features such as the cotyledon, radicle, and endosperm were defined. An average spectrum across the spectra of the group of features was used for the definition of peak integration ranges, based on the average peak intensities obtained for each group. Then, orthogonal partial least squares discrimination analysis (OPLS-DA) was performed by SIMCA 14.1 (https://www.sartorius.com/en/products/process-analytical-technology/data-analytics-software/mvda-software/simca) for differentiating samples with different quality parameters and discovering the potential quality-associated markers.

### Supplementary Information

Supplementary Information.

## Data Availability

The original contributions presented in the research are included in the article/Supplementary Material. Further inquiries can be directed to the corresponding authors.
