# Supplementary material for: Spatial distribution of metabolites in processing Ziziphi Spinosae Semen as revealed by matrix-assisted laser desorption/ionization mass spectrometry imaging
Source: Sci Rep. 2024 Jul 3;14:15263. doi: 10.1038/s41598-024-61500-w (PMC11222422; doi:10.1038/s41598-024-61500-w)
Supplement: Supplementary file 1 — Supplementary Information. [file 41598_2024_61500_MOESM1_ESM.pdf]

Supplementary material

Spatial Distribution of Metabolites in Processing Ziziphi Spinosae Semen as Revealed by Matrix-Assisted Laser Desorption/Ionization Mass Spectrometry Imaging

Donglai Ma[1,2,3†], Mengwei Zhao[1†], Haochuan Guo[1], Lili Wang[4*], Yage Li[1], Shinong Yuan[1], Yuping Yan[1,2], Yuguang Zheng[1], Xian Gu[1], Yongxing Song[1], Xiaowei Han[1*], Huigai Sun[1,2*]

[1]School of Pharmacy, Hebei University of Chinese Medicine, Shijiazhuang 050200, China.

[2]Traditional Chinese Medicine Processing Technology Innovation Center of Hebei Province, Shijiazhuang 050200, China.

[3]International Joint Research Center on Resource Utilization and Quality Evaluation of Traditional Chinese Medicine of Hebei Province, Shijiazhuang 050091, China.

[4]College of Chemistry and Chemical Engineering, Xingtai University, Xingtai 054001, China.

*Corresponding author: 201820366@xttc.edu.cn (Lili Wang); hanxiaowei2015@126.com (Xiaowei Han); Sunhuigai66@163.com (Huigai Sun)
[†]These authors have contributed equally to this work.

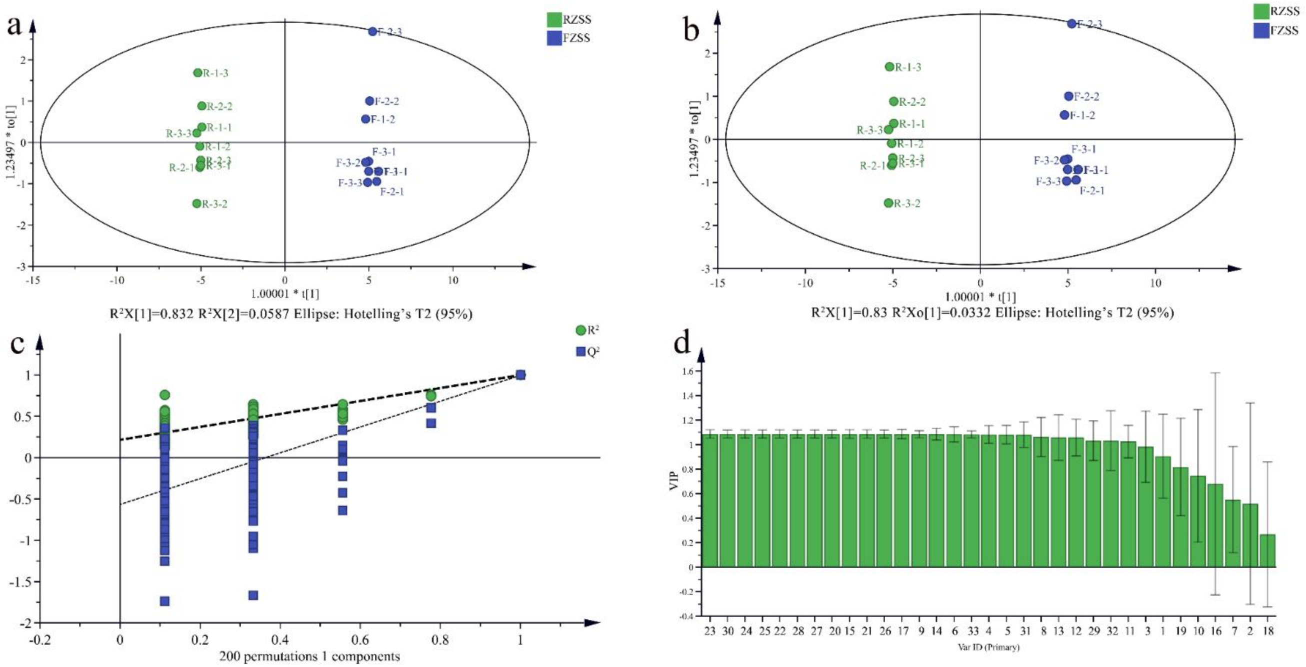

Supplementary Figure S1. PCA score plot (**a**), OPLS-DA score plot (**b**), permutation test plot (**c**), and VIP value (**d**) of the potential quality-associated markers for RZSS and FZSS discovered.

Supplementary Table S1. Identification of differential metabolites in RZSS and FZSS tissue sections using MALDI-MS/MS.

No.	Metabolite	Formula	Ion formula	Theoretical *m/z*	Observed *m/z*	Ion abundance		Relative difference (%)	fragment ions *m/z*
						RZSS	FZSS		
1	Jujuboside A	$C_{58}H_{94}O_{26}$	$[M+K]^+$	1207.35	1245.566	2635.56±116.79	3126.44±218.80	−18.63	927, 603, 473, 279, 67
2	Jujuboside B	$C_{52}H_{84}O_{21}$	$[M+H]^+$	1045.21	1045.558	565±29.80	538.22±21.88	4.74	942, 763, 455, 305, 119, 39
3	Sanjoinenine	$C_{29}H_{35}N_3O_4$	$[M+H]^+$	489.67	490.270	918±41.23	1282.89±123.02	−39.75	242；131
4	Spinosin	$C_{28}H_{32}O_{15}$	$[M+K]^+$	608.54	647.137	432.55±8.95	1149.33±64.06	−165.72	351, 297
5	6'''-Feruloylspinosin	$C_{38}H_{40}O_{18}$	$[M+H]^+$	784.71	785.228	56340.89±1653.16	85410.78±1737.27	−51.60	327, 351, 177, 297, 429, 665, 785
6	AGOME	$C_{22}H_{22}O_{10}$	$[M+Na]^+$	446.44	469.111	456.33±11.28	822.78±20.36	−80.32	--
7	Vicenin-2	$C_{27}H_{30}O_{15}$	$[M+Na]^+$	594.5	617.147	5800.44±153.47	6000.22±198.76	−3.44	577, 385, 309, 163, 89
8	6''-p-Coumaroylspinosin	$C_{38}H_{40}O_{17}$	$[M+Na]^+$	768.78	791.216	777.22±22.57	1040.89±38.36	−33.92	447, 429, 351,
9	6'''-Sinapoylspinosin	$C_{39}H_{42}O_{19}$	$[M+H]^+$	798.76	799.244	3710.78±89.95	11178.11±361.53	−201.23	635, 447, 429, 393, 351, 327, 297, 207

No.	Metabolite	Formula	Ion formula	Theoretical *m/z*	Observed *m/z*	Ion abundance		Relative difference (%)	fragment ions *m/z*
						RZSS	FZSS		
10	CMBDPA	$C_{30}H_{42}N_4O_5$	$[M+H]^+$	538.7	539.323	2380.56±76.71	2055.79±306.76	13.64	--
11	Nuciferine	$C_{19}H_{21}NO_2$	$[M+H]^+$	295.4	296.164	784.23±20.22	891.44±19.85	−13.67	265, 296, 250; 234; 237
12	Amphibine D	$C_{36}H_{49}N_5O_5$	$[M+H]^+$	631.9	632.381	546.55±11.00	737.22±30.47	−34.89	148, 289, 261,316, 344, 632
13	N-nornuciferine	$C_{18}H_{19}NO_2$	$[M+Na]^+$	281.38	304.131	1409.78±9.87	1485.33±8.08	−5.36	--
14	Palmitic Acid	$C_{16}H_{32}O_2$	$[M+H]^+$	256.42	257.247	917.67±2.58	1062.67±5.52	−15.80	153, 83, 27
15	Oleic acid	$C_{18}H_{34}O_2$	$[M+H]^+$	282.46	273.185	3991.89±11.65	1883±7.26	52.83	69, 41
16	Succinic Acid	$C_4H_6O_4$	$[M+Na]^+$	118.088	215.016	725.56±28.95	698.44±2.71	3.74	73, 27
17	Citric acid	$C_6H_8O_7$	$[M+K]^+$	192.13	230.990	1907.56±4.57	845.11±15.55	55.70	94, 72, 70
18	3-O-trans-p-coumaroyl maslinic acid	$C_{39}H_{54}O_6$	$[M+K]^+$	618.843	657.355	636.22±22.82	627.44±13.91	1.38	551, 433, 289, 179, 119
19	Ceanothic acid	$C_{30}H_{46}O_5$	$[M+Na]^+$	486.683	509.324	729.89±31.38	774.22±33.23	−6.07	289, 167, 67
20	Butanedioic acid	$C_4H_6O_4$	$[M+H]^+$	162.14	163.060	1592.29±1.65	2050.08	−28.75	100, 74, 55
21	Lauric acid	$C_{12}H_{24}O_2$	$[M+H]^+$	200.3178	201.185	3570.5.±1.14	2969.40±2.98	16.84	--

No.	Metabolite	Formula	Ion formula	Theoretical *m/z*	Observed *m/z*	Ion abundance		Relative difference (%)	fragment ions *m/z*
						RZSS	FZSS		
22	Glutamic acid	$C_5H_9NO_4$	$[M+K]^+$	147.13	186.016	13650.33±1.41	12160.11±4.51	10.92	85, 56, 42
23	Proline	$C_5H_9NO_2$	$[M+Na]^+$	115.13	138.052	2843.78±1.47	2222.89±2.02	21.83	70, 68
24	Lysine	$C_6H_{14}N_2O_2$	$[M+K]^+$	146.19	185.069	22206.56±4.30	19857.56±3.44	10.58	84, 56
25	Phenylalanine	$C_9H_{11}NO_2$	$[M+H]^+$	165.19	166.086	14506.89±2.28	9955.67±2.26	31.37	166，120，103，91
26	Methionine	$C_5H_{11}O_2NS$	$[M+Na]^+$	149.21	172.040	2619.00±2.11	2234.22±3.61	14.69	104, 56
27	L-Isoleucine	$C_6H_{13}NO_2$	$[M+K]^+$	131.17	170.058	164236.10±1.79	151610.6±4.88	7.69	70, 57, 41
28	Arginine	$C_6H_{14}N_4O_2$	$[M+H]^+$	174.2	175.119	2407.78±1.47	19526.33±1.89	−710.97	70, 43
29	Serine	$C_3H_7NO_3$	$[M+H]^+$	105.09	106.050	715.78±26.18	606.11±1.52	15.32	88, 46
30	(S)-Coclaurine	$C_{17}H_{19}NO_3$	$[M+K]^+$	285.34	324.099	2675.44±13.98	4570.44±25.48	−70.83	257, 121, 79
31	Eleutheroside A	$C_{35}H_{60}O_6$	$[M+H]^+$	576.95	577.446	1147.56±45.26	1858.22±63.35	-61.93	--
32	Adenosine	$C_{10}H_{13}N_5O_4$	$[M+K]^+$	267.24	306.060	863.56±17.22	952.56±12.77	-10.31	136, 119, 94, 92, 57
33	Catechin	$C_{15}H_{14}O_6$	$[M+H]^+$	290.27	291.086	3253.78±11.22	3470.44±6.27	-6.66	259, 123, 51
